# Multiple In Vitro Analyses of Fracture Resistance in Maxillary Central Incisors Restored with Fiber Posts

**Published:** 2010-08-15

**Authors:** Fariborz Vafaee, Masoumeh Khoshhal, Aliakbar Rezaei, Fereidon Sooltani, Mohsen Jalalzadeh, Ali Yalpaniyan, Farnaz Firooz, Ali Reza Izady, Ebrahim Yarmohamadi

**Affiliations:** 1. Department of Prosthodontics, Dental School/Dental Research Center, Hamadan University of Medical Sciences, Hamadan, Iran.; 2. Department of Periodontics, Dental School, Hamadan University of Medical Sciences, Hamadan, Iran.; 3. Department of Prosthodontics, Dental School, Shahid Beheshti University of Medical Sciences, Tehran, Iran.; 4. Department of Endodontics, Dental School, Hamadan University of Medical Sciences, Hamadan, Iran.; 5. Mechanical Engineer MSc, Tehran, Iran.; 6. Department of Prosthodontics, Dental School, Hamadan University of Medical Sciences, Hamadan, Iran.; 7. Department of Restorative Dentistry, Dental School, Hamadan University of Medical Sciences, Hamadan, Iran.

**Keywords:** Finite Element Analysis, Fiber Posts, Incisor, Photo Elastic Analysis, Tooth Fractures

## Abstract

**INTRODUCTION:**

The resistance to fracture of endodontically treated teeth restored with esthetic post systems has not been extensively researched. This in vitro study compared the fracture patterns of endodontically treated teeth with esthetic post systems with different analysis methods.

**MATERIALS AND METHODS:**

A total of 26 recently extracted human maxillary central incisors were decoronated and then endodontically treated. Teeth were restored with quartz fiber posts. All posts were cemented with Panavia dual curing adhesive resin cement and subsequently restored with composite cores. Three methods were used to test fracture resistance. Each specimen was embedded in acrylic resin and then secured in a universal load-testing machine. A compressive load was applied at 135º degree angle at a crosshead speed of 1 mm/min to the long axis of the tooth until fracture occurred. The two other methods, finite element analysis (FEA) and photo elastic study used the same angulation and 90 N force to simulate the first method. The data were then compared.

**RESULTS:**

Clinical results indicated that fracture was most likely to occur between core and dentin, and then in the cervical 1/3 of the root. Photo elastic study demonstrated similar results; the highest stresses occurred at the junction of dentin and core contralateral to the side where force was applied. FEA also confirmed these results; however it also showed that the highest stresses arise at the dentin/core junction contralateral to the force point.

**CONCLUSION:**

All three techniques reiterate that the risk of fracture is greatest at the cervical dentin/core junction.

## INTRODUCTION

Endodontically treated teeth are generally weakened due to the loss of sound structure from caries, previous restorative procedures and endodontic access cavity preparation. To prevent further destruction of these teeth, a protective restoration is necessary to create retention and resistance [1].

A widely used method for the treatment of structurally weakened teeth is the post and core systems. The primary objective of post and core procedure is replacement of the lost tooth structures in order to facilitate crown support and retention [2]. There are various post and core systems. The most widely used systems can be classified into two basic types, metal posts and cores that are custom cast as a single piece, and two element designs comprising a prefabricated post to which a silver amalgam or composite core is subsequently adapted. The construction of post-core castings is relatively more time consuming and demands extra clinic and laboratory time, in addition to increased costs [3]. Prefabricated posts are faster, cheaper and easier to insert in the restoration of endodontically treated teeth [4][5].

Stress distribution analysis of prefabricated post applications have been studied by many researchers using different theoretical or experimental techniques [6][7][8][9]. Fiber posts show more homogeneous stress distribution than metallic posts [10][11]. Fiber posts can be recommended as an alternative to cast and prefabricated metallic posts [12]. Yamamoto et al. suggested that abutment build-up using composite resin core in combination with a glass fiber post model produced the lowest stress [13].

Photo elastic technique visually demonstrates the stress distribution around structures. This technique is a relatively qualitative visual measurement based on the ability of certain transparent materials to exhibit interference fringes when stressed in a field of polarized light. The distinct fringes illustrate zones of stress intensity and concentration and can be identified as a sequence of colored bands [14].

Finite element analysis (FEA) has become a popular numerical method in stress analysis and has been applied to dental biomechanics for the last decades [15][16]. FEA method is based on a mathematical model which approximates the geometry and the loading conditions of the structure to be analyzed. Deformation and stress distributions in response to different loading conditions can be simulated with the aid of computers and the most stressed areas can thus be evidenced [17]. The purpose of this study was to evaluate the effects of fiber post restoration on the stress distribution in maxillary central incisor with three different techniques in vitro fracture test, photo elastic and finite element. The null hypothesis was that there was no difference between the clinical data, FEA and photoelastic method.

## MATERIALS AND METHODS

### Part 1. Clinical method

Twenty six central incisors extracted during the past 3 month with relatively similar sizes were selected. They were disinfected and debridement with scalers (Hu-Fredy, USA) and subsequently stored in normal saline. Root canal therapy was conducted using step back technique until master apical file (MAF) size 45 was reached (Mani, Nakaakutsu, Japan); the crown was evenly reduced from 2 mm above the CEJ and gutta-percha removed coronally, leaving 4mm of gutta-percha at the apical third of root canal with #3 RTD burs (RTD, Grenoble, France). Specific post drill for light post (RTD, Grenoble, France) size #3 was used to shape the canal. Root canal was etched with 37% phosphoric acid (Ultradent, USA) for 10 second and then rinsed with water for 30 seconds. Panavia F 2.0 dual cure primer (Kurary, Japan) was mixed and inserted into the canal; after 30 second excess was removed with paper points (Ariadent, Iran). Panavia F 2.0 dual cure cement pastes (Kuraray, Osaka, Japan) were mixed and applied to the post; the post was then inserted into canal and light cured for 30 second. Core buildup was carried out with the aid of crown former no 21 (Shady dental, Tehran, Iran) and photocore composite resin (Kuraray, Osaka, Japan) with 4.8 labiolingual. The tooth was then fixed in self cure acrylic resin (Bayer. AG, Germany). The tooth was then fixed in self cure acrylic resin (Bayer. AG, Germany) and a jig for testing the machine (Instron, Iran) was fabricated to fix the tooth with 45° angle to the force [18][19][20]. The testing machine used 1 mm/min speed to fracture the tooth; the force was aimed at the upper part of the tooth cinglum. After fracture, the maximum load and the pattern of fracture were assessed.

### Part 2. Photoelastic modeling

Three blocks of PSM-5 photoelastic material (Measurements groups inc., Raleigh, NC, USA) were prepared with 10×45×45 mm dimensions. PSM-5 is an epoxy resin with high elastic modulus similar to that of human dentin, good stress-optic and creep properties [6][15][21]. The prepared blocks were tested by a polariscope (Photolastic Inc., Raleigh, USA) to ascertain that they were free of residual stresses. An artificial canal 10 mm in length and 0.8 mm in width was prepared 90 degrees to the block edge by a press drill instrument (Superstar Co, China). The canals were prepared for DT Light- post (RTD, France) size #3 according to manufacturer instructions. External canal portion of post was reduced to 4.5 mm. Posts were cemented in the canals by Panavia F cement (Kurary Dental, Japan) according to manufacturer’s instructions. The build-up core on the posts was constructed with Photocore composite resin with 4.8 mm labio-lingual and 5.8 mm bucco-lingual dimensions in the cervical region and 4.5 mm in the vertical height.

All specimens were tested and photographed by polariscope with and without color filter in following situations:

-without loading

**Figure 1:**
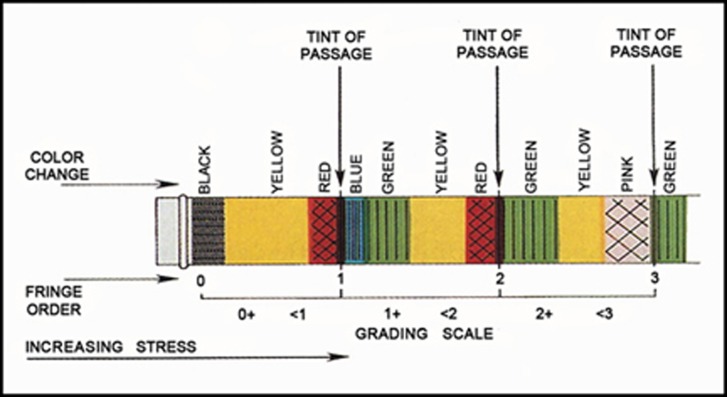
Illustration of fringe orders and color changes in photoelastic stress analysis

-with 90 N [21][22] oblique load; i.e. force exerted 45 degrees [23][24] to the long axis of the posts. The photographs were analyzed and the Fringe orders were determined in the coronal, middle and apical third of the posts: in order to determine the fringe order, pattern illustrated in Figure 1 was used [21].

Difference in fringe order equal to or greater than one was assumed as significant difference.

### Part 3. finite element model

The three-dimensional (3D) model of maxilla was prepared based on a CT scan projection of a real patient. The internal border of the cortical bone was defined in Photoshop CS4 environment (Adobe Systems Incorporated, San Jose, CA) for several sequential sections. The final 3D model of the maxilla was formed using Solid Works (Solid Works Corp., Concord, MA, USA) and NURBS (McNeel, Seattle, USA) software. Due to the resilience of the soft tissue, the contact area of the tooth and soft tissue was assumed to be frictionless. To analyze final models, different elements were first assembled in the forms of maxillary central incisor reinforced with fiber post composite core and then inserted into ANSYS Workbench platform 2.0 (ANSYS Inc., Osaka, Japan). Tetrahedral elements were used for mesh (grid) generation. Smaller elements were generated where higher precision was needed. Static analysis of the models was also performed in the ANSYS.

Mechanical properties of the simulated materials are presented in Table 1.

**Table 1:** Material properties

**Material**	**Elastic Modulus Gpa**	**Poisson Ratio**
**Cancellous bone**	1.37	0.3
**Cortical bone**	13.7	0.3
**Dentin**	18	0.31
**PDL**	0.5	0.45
**Panavia**	18.6	0.28
**Photo core kurary**	18.6	0.26
**Gutta-percha**	0.69	0.45
**Fiber post**	20	0.28

The loads were exerted to simulate the natural contact of lower incisor to upper part (incisal) of the upper central incisor with 90 N force and 45 degree.

## RESULTS

### Part 1. clinical results

**Figure 2:**
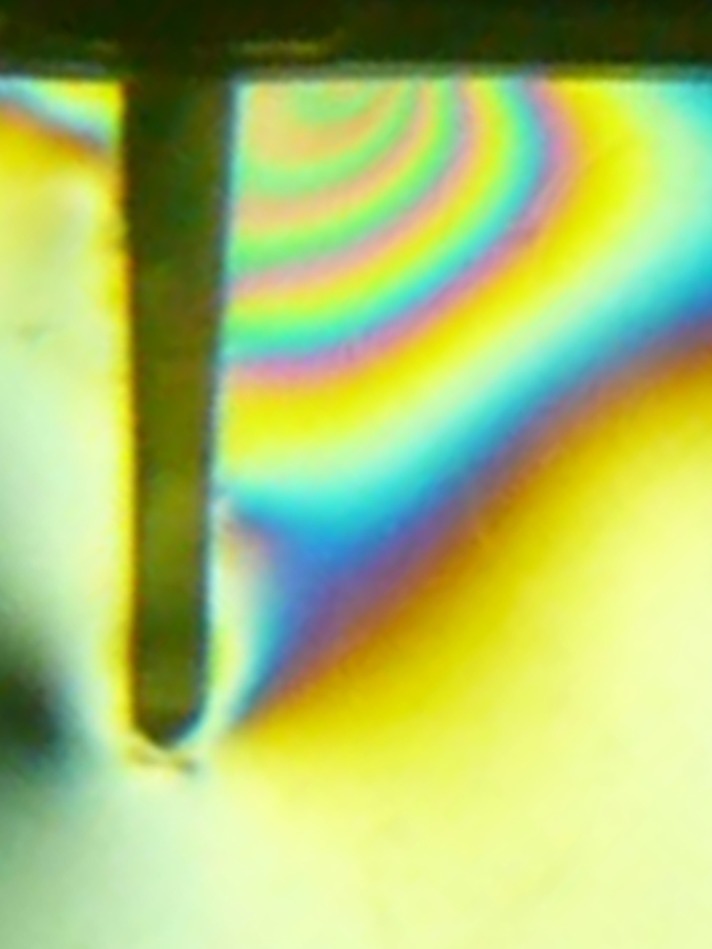
Teeth fracture pattern

Clinical fracture test was conducted first; the maximum mean fracture at 45 degrees and 1 mm/min speed was 293±37 N. The fracture patterns are shown in Figure 2. Majority of fractures are between the core and root, and then in the cervical third of the root.

### Part 2. photo elastic results

**Figure 3:**
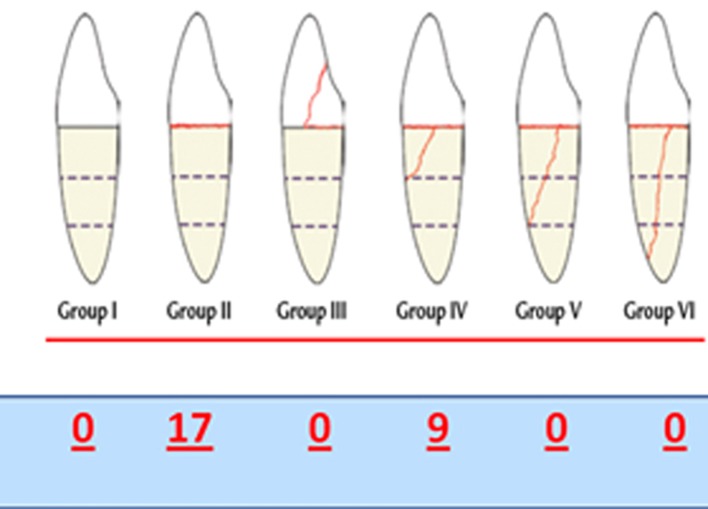
Photo elastic

In the absence of loading, all specimens were stress free. Under oblique loading (Figure 3), all specimens had identical stress distribution patterns. Higher stress concentration was seen in the cervical region at the opposite side of loading (FO=6). Cervically, on the same loading side, fringe order 2 was observed (FO=<2). In the apical region, lower stress concentration was recorded with FO=<2.

### Part 3. finite element results

**Figure 4:**
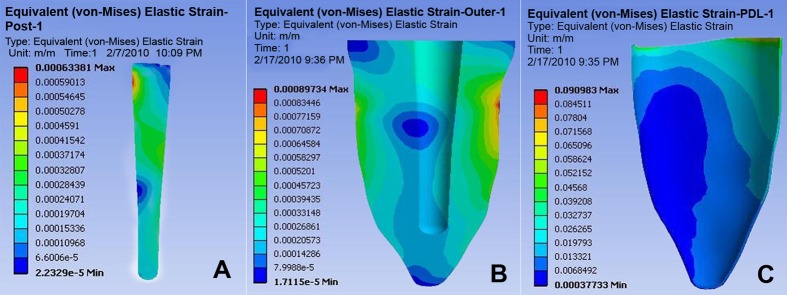
A) Fiber post (FEM). B) Dentine (FEM). C) Pdl (FEM)

**Figure 5:**
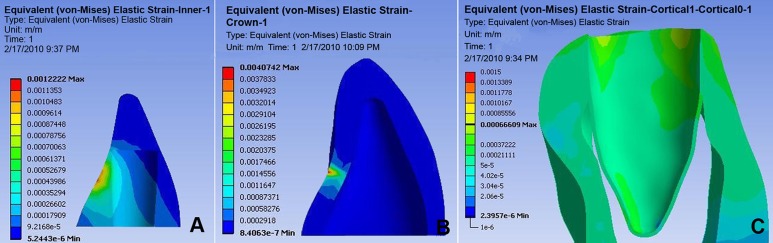
A) Core material (FEM). B) Crown (FEM). C) Cortical bone (FEM)

FEM strain distribution results in dentin illustrate that the highest compressive strains lie in the cervical third, when tooth is in contact with bone and opposite to the force point (i.e. buccal) as well as underneath the core at the palatal side (tensile). The lowest strain areas were found in the mesial and distal middle part of the tooth root and also in the region of palatal dentin edge (Figure 4-Figure 5).

At bone level two tension areas can be witnessed bucally (junction of tooth and bone) and one compression area can be seen apically in the palatal one third. At the cervical palatal region there is an area under tensile force. The highest level of strain can be demonstrated at the palatal junction of the core material with dentin.

## DISCUSSION

Anterior teeth, especially maxillary central incisors, disclude and protect posterior teeth through protrusive movements as well as tear foods during mastication. The stresses introduced during tearing must be considered when determining the long-term success of restorations. An endo-crown is a full or compact crown that extends a post into the pulp chamber and/or pulp canals as one homogenous unit rather than as several units of post core and crown [25]. Stress concentrates where a non-homogeneous material distribution is present, such as the interface regions. The interfaces of materials with different module of elasticity represent the weak point of a restorative system, as the toughness/stiffness mismatch influences the stress distribution [26][27].

In many applications, a system is subjected to the repeated application of a stress below the yield strength of the material. Even though the stress is below the yield strength, the material may fail after a large cumulative number of applications of stress. Failure occurs by a process involving nucleation of a crack, slow propagation of the crack, and subsequent catastrophic failure of the system. Cracks nucleate at locations of highest stress and lowest local strength. Dental restorative systems investigated either under static or fatigue loading conditions showed similar failure patterns [28][29][30].

A static linear analysis can be successfully applied to extrapolate reliable information about the relative susceptibility of systems to fatigue loading conditions. Fatigue loading is the most common form of stress in the oral environment; this form of loading emphasizes stress arising in critical areas which causes systems to fail [31]. Therefore, our assumption is that systems showing homogeneous stress distribution in a static analysis (better stress distributing capability) would illustrate less fatigue sensitivity in the clinical applications. The present study was designed to compare stress distribution in post restored teeth with different analysis techniques in order to identify areas of high stress concentration, where fatigue failures are more likely to occur [32]. As a consequence, attention was mainly focused on the overall stress distribution arising in a maxillary central incisor where the crowns were lost. Three different analyses were tested in order to evaluate which more closely simulates the biomechanical behavior of a sound tooth and provides more information about stress distribution. It is well known that the clinical analysis is the most realistic one; however it does not provide further information like the two other techniques. FEA offers rich and detailed data with regards to fracture probability; however it must be compared with clinical data and photo elastic analysis.

FEM show stress in decreasing order 1) buccocervical third 2) apicopalatal third 3) middle buccal third; this concurs with previous studies [33][34][35]. This stress pattern is comparable to the clinical results; that is most fractures occur at the junction of the core and post. Moreover, both photo elastic and FEM analysis agree with clinical data that the most stressful area is at the post/core material/dentin junction (Figure 5). Fem showed higher stresses in the cervical palatal third of the post; very similar results were reported by others, in a direction that suggested deboning of the post [36][37].

FEM showed that the greatest strain occurred on the palatal side; however, photo elastic method demonstrated greater strain in the buccal area. This may be due to the lower detailed data obtain from the photo elastic method or the incomplete bonding of core material palatally.

## CONCLUSION

Data gathered from the three techniques indicated that the most probable initial point of fracture is at junction of dentin/core material.

All of the three techniques estimated where fracture may begin; however, the clinical method was the most costly, time consuming and not particularly hygienic. Photo elastic method provides more data about stress patterns; however its drawbacks are that it required many models for analysis of dentine/bone/PDL/post/core. Moreover it was costly and time consuming and also did not simulate fracture or estimate where the fractures initiated. The finite element method, on the other hand, provides more detailed data and as it is based on mathematical data and analysis, it has more accurate information. Furthermore, it can predict where the fracture may occur.
